# Spatial and temporal resolutions of EEG: Is it really black and white? A scalp current density view

**DOI:** 10.1016/j.ijpsycho.2015.05.004

**Published:** 2015-09

**Authors:** Borís Burle, Laure Spieser, Clémence Roger, Laurence Casini, Thierry Hasbroucq, Franck Vidal

**Affiliations:** a Aix-Marseille Université, CNRS, LNC UMR 7291, 13331 Marseille, France; b SCALab, UMR CNRS 9193, Université de Lille, France

**Keywords:** EEG, Current Source Density, Time resolution

## Abstract

Among the different brain imaging techniques, electroencephalography (EEG) is classically considered as having an excellent temporal resolution, but a poor spatial one. Here, we argue that the actual temporal resolution of conventional (scalp potentials) EEG is overestimated, and that volume conduction, the main cause of the poor spatial resolution of EEG, also distorts the recovered time course of the underlying sources at scalp level, and hence degrades the actual temporal resolution of EEG. While Current Source Density (CSD) estimates, through the Surface Laplacian (SL) computation, are well known to dramatically reduce volume conduction effects and hence improve EEG spatial resolution, its positive impact on EEG temporal resolution is much less recognized. In two simulation studies, we first show how volume conduction and reference electrodes distort the scalp potential time course, and how SL transform provides a much better spatio-temporal description. We then exemplify similar effects on two empirical datasets. We show how the time courses of the scalp potentials mis-estimate the latencies of the relevant brain events and that CSD provides a much richer, and much more accurate, view of the spatio-temporal dynamics of brain activity.

## Highlights

• We investigated the impact of volume conduction on the temporal resolution of EEG.
• Simulations revealed a degraded temporal resolution of scalp potential data.
• Current Source Density allows to nicely recover the underlying time courses.
• These effects are shown on two empirical datasets.
• The importance of deblurring methods, such as CSD, is discussed.

## 1 Introduction

In the last fifteen years, our understanding of the brain–behavior relationship has dramatically improved, largely thanks to brain imaging techniques (see below). Looking at “the brain in action” while it performs on some tasks allows a direct assessment of its functioning properties. This also allows to better constraint functional, cognitive, models. Indeed, in addition to account for behavioral performances, models must also account for additional, intermediate, cerebral indices. For example, the presence of an activity in region *R* supposed to implement a function *F* may inform us about the underlying processes involved in the task at hand (with some necessary precautions, though, see Poldrack, 2006; Vidal et al., 2015). Similarly, the relative timing of two regions *R*_1_ and *R*_2_, and hence of functions *F*_1_ and *F*_2_ can provide essential clues about the underlying architecture.

Non-invasive brain imaging techniques usable in humans fall into two main families: metabolic-based (functional magnetic resonance imaging — fMRI, positron emission tomography — TEP, near infrared spectroscopy — NIRS, etc.) and electrophysiological-based (mainly electro- and magneto-encephalography).^1^ Metabolic techniques are classically considered as having a very good spatial “resolution”, but a rather poor temporal one, while electrophysiological techniques are assumed to have an *excellent* temporal resolution, and a poor spatial one.

**Fig. 1:**
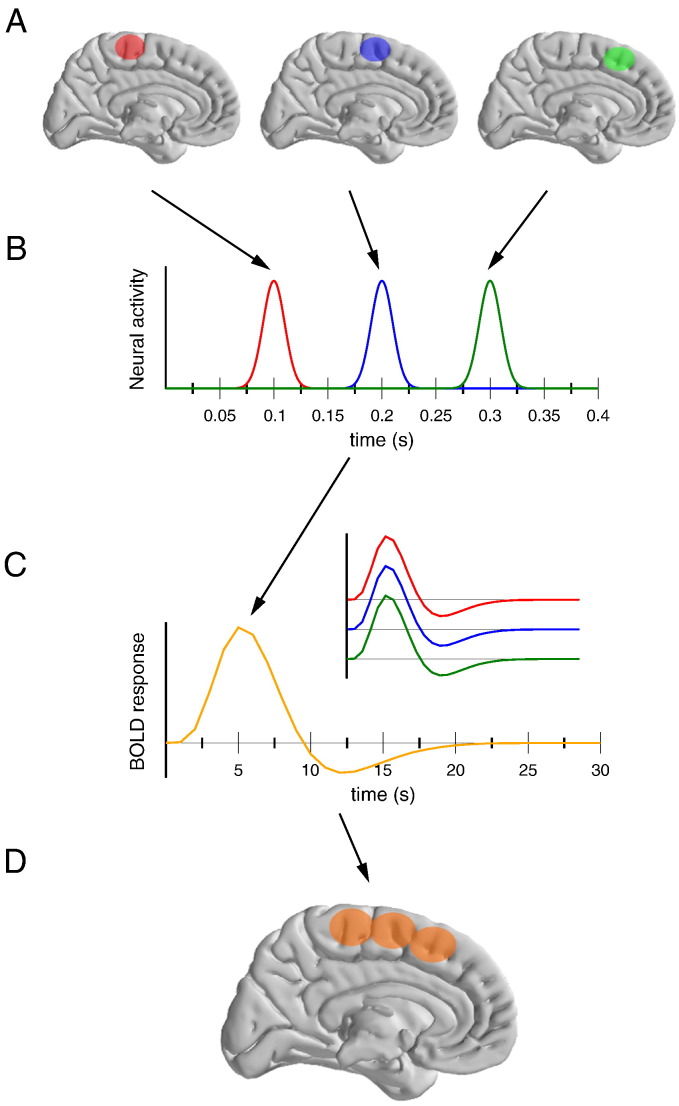
Schematic representation of the interaction between temporal and spatial resolutions in brain imaging studies, for example fMRI. Let's assume three neighboring regions (panel A) that are activated independently and sequentially (panel B). Their corresponding BOLD response will be identical (panel C). As a consequence, the activations of these three regions will not be separable. The resulting activation pattern (panel D) is much less refined than the actual activated areas (panel A), hence degrading the actual spatial resolution of the technique.

These different techniques are classically plotted in bi-dimensional maps, with the two axes being these two resolutions (see e.g. Sejnowski and Churchland, 1990; Walsh and Cowey, 2000). Plenty of such representations can be found in the literature with very small differences, but they all share a common feature: the two dimensions, i.e., the spatial and temporal resolutions, are, more or less implicitly, assumed to be independent, that is the spatial resolution of a given technique is independent from its temporal one, and vice versa. For this reason, the two resolutions are represented on orthogonal axes. Although true from a technical point of view, this idea may not hold from the psychologist's or neuroscientist's point of view. Indeed, for who is interested in understanding brain processes, the “resolution” of a technique corresponds to the minimal step (in space, or time) at which separated cerebral activities could be observed. From this point of view, independence between temporal and spatial resolutions is far from being warranted, and we will argue that a phenomenon lowering one of the two resolutions often degrades the other one. It is easy to illustrate how the low temporal resolution of metabolic techniques may mask temporally separated activations into a single, more spread activity. Fig. 1 presents a simple cartoon scenario that illustrated this interdependence for metabolic imaging (fMRI, PET etc.). Let's assume that three adjacent areas (panel A) are active sequentially, with a small delay (panel B). The slow time course of the BOLD signal leads to an identical haemodynamic response for the three areas (panel C and inset).^2^ These three areas showing the very same response, they will not be separable, and the resulting activation will be the sum of the three areas. The spatial extend of the recovered activation is hence much larger than the real anatomical activation, degrading the actual spatial resolution of the measure. In this example, the impossibility to temporally separate the different activations degrades the spatial resolution of the technique. As we will show below, the symmetric reasoning also holds true for EEG and the factors responsible for its poor spatial resolution also limit its actual temporal one.

### 1.1 Bad spatial but good temporal resolution?

In human participants, scalp electrodes are classically used to record brain electrical activity, that is electrical events generated several centimeters below the recording electrodes. As a consequence, cortical current must go through different resistive layers which provide at scalp level a distorted view of the brain activities (Nunez et al., 1994). More specifically, those various layers, and especially the skull (Srinivasan et al., 1996), induce a blurring effect at scalp level. As a consequence, at every spatial scalp position, the recorded activity is a mixture (i.e. a weighted sum) of the underlying brain sources (Makeig et al., 1996). Such volume–conduction-induced mixture is the main cause of the poor spatial resolution of scalp EEG (around 5 to 9 cm, Nunez et al., 1994; Babiloni et al., 2001). In addition, the necessary use of a reference electrode to measure difference in potential also contributes to this spatial smearing. The volume conduction effect on EEG spatial resolution has largely been discussed and described, and readers are referred to relevant literature (see e.g. Nunez and Westdorp, 1994; Tenke and Kayser, 2012 for gentle introductions, and Nunez and Srinivasan, 2006 for more complete discussion). Introduction of “high resolution EEG” techniques (Gevins, 1993), among which surface Laplacian (SL) has played a critical role (Nunez et al., 1994; Babiloni et al., 1995), allowed to dramatically improve the spatial resolution of EEG.

While it is largely acknowledged and widely accepted that volume conduction and reference electrode deteriorate spatial resolution of scalp EEG, other distortions are less widely recognized in the community. As a matter of fact, the time course of brain activities is also largely distorted. For example, spontaneous EEG signals recorded by different electrodes tend to appear more phase locked than they actually are, inducing artifactually high between site coherence (Nunez et al., 1997). In what follows, we will show how the timing of averaged event-related potentials (ERPs) is also altered by the same factors. This degraded temporal resolution is seldom acknowledged in the literature (see Law et al., 1993 for an exception), and it is still widely assumed that the timing of scalp potential provides an accurate timing of the underlying sources, since electrical activity propagates instantaneously to the recording electrodes. However, the mixture induced by the spatial smearing also temporally mixes the underlying activities hence making the scalp potential temporal resolution significantly lower than usually assumed. Importantly, we will show that techniques improving the spatial resolution of scalp EEG also secondarily largely improve the temporal one.

Different methods have been proposed to increase the spatial resolution of EEG, that differ in their computational complexity and physiological and physical assumptions. In the present report, we will focus on the SL transform or Current Source Density (CSD). The SL of the scalp potential being proportional to the flow of current entering the inner skull allows to get rid of the skull-induced volume conduction, and hence provides a fair estimate of the corticogram (see Tenke and Kayser, 2012 or Giard et al., 2014 for recent reviews and presentations). Note that, theoretical studies have argued that CSD is poorly sensitive to deep sources (Pernier et al., 1988). From an empirical point of view, its real (un)sensitivity still needs to be deciphered. Indeed, empirical reports suggest that no information is lost by after applying CSD transform (Kayser and Tenke, 2006a). Furthermore, this sensitivity to deep sources might well be function of computation parameters (see Kayser and Tenke, 2006a; McFarland, 2015). While the dramatic improvement of EEG spatial resolution brought by SL transform is widely acknowledged and is now undisputed (see Nunez and Westdorp, 1994), its role in temporal resolution improvement is much less recognized. We will argue here that a good temporal resolution can only be achieved if a fair spatial resolution is reached, showing the inter-dependency of the two dimensions. We will also argue that SL transform allows a very good improvement of both dimensions, at low computational costs and with few necessary assumptions. We will first present two simulation studies in which we will compare the timing of the scalp potential and the SL (at scalp level) with the timing of the simulated cortical sources. After illustrating such temporal distortion effects on simulation, we will then show similar effects in two empirical datasets.

## 2 Impact of volume conduction on the scalp activity timing: simulation studies

The impact of spatial blurring on the temporal property of the signal recorded at scalp level will first be illustrated and demonstrated through simulated data: scalp potentials generated by cortical dipoles, whose characteristics will be manipulated, will be computed, and we will examine how the variations of dipole activities are reflected in both reconstructed scalp potentials and SL data.

### 2.1 General simulation method

#### 2.1.1 Head and forward models

**Fig. 2:**
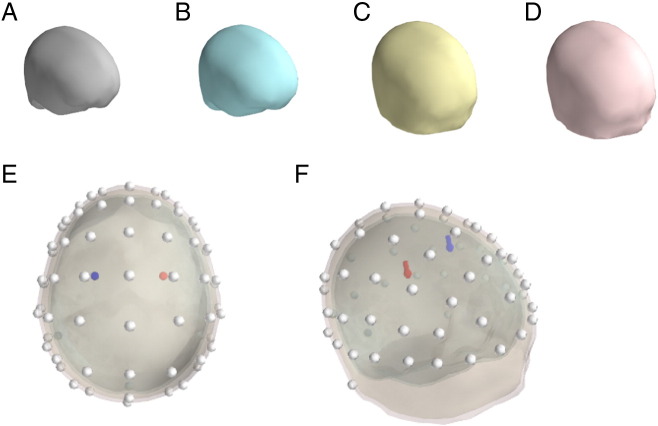
Simulation model. A–D. Graphic representation of the four meshes used as interface. A. Interface between CSF and brain. B. Between CSF and inner skull. C. Between outer skull and scalp. D. Between scalp and air. E–F. Location of the simulated dipoles, represented inside the head model. E. Top view. F. Lateral view.

The head model used here was based on a segmentation of the MNI152 template brain and the leadfield and forward solution were computed with the OpenMEEG software (Gramfort et al., 2010; Kybic et al., 2005), which is based on a symmetric Boundary Element Method. Four nested layers were modeled (Fig. 2A–D): the brain envelope (smoothed outer part of the brain), the cerebro-spinal fluid (CSF), the outer skull and the scalp. The conductivity of air was set to 0. The other conductivities are relative, and the conductivity of the skull was set to .03 of the brain, while the CSF conductivity was set to 3 (3 times the brain and skin one, both set to 1). Sixty-four electrodes were modeled, located on the standard extended 10–20 system positions (Fig. 2E–F).

#### 2.1.2 Source modeling

For the two simulations, two symmetrical equivalent dipoles were positioned into the cortical volume at positions x = ± 30 mm, y = 0 mm and z = 50 mm in the standard MNI space. As shown in Fig. 2E–F, the two dipoles were located approximately below electrodes C1 and C2. Both dipoles were oriented vertically (orientation: 0, 0, 1). Dipole time courses were modeled as Gaussian curves (see below for details for each simulation). For each simulation, the dipole activities (simulated sample interval: 1 ms, that is 1000 Hz) were projected onto the electrodes at every time point, through the leadfield computed as described above, giving measures of what one would get with conventional scalp potential (up to an additive constant). Different reference electrode configurations were implemented: the scalp data were referenced to electrodes located over the left mastoid, the right mastoid, the nose and the (off-line) linked mastoids.

#### 2.1.3 Data processing

The time courses of the reconstructed scalp potential on each electrode and for each reference electrode were analyzed as one would do with real EEG measures. In a second step, the data were SL transformed. This was done following Perrin et al.'s (1987, 1989) method, as implemented in the CSD toolbox (Kayser and Tenke, 2006b). Note, however, that we used a re-implementation of the algorithm in Python. The order of spline used was set to 3 (*m* parameter in Kayser and Tenke, 2006b), and the smoothing constant was set to 10^− 5^ (*λ* parameter). From the potential, the SL was computed on all electrodes and at all time points.

For the sake of simplicity, analyses were focused on the central electrodes (C1, Cz and C2), above the simulated sources. The latencies of the peak of activity for these electrodes were extracted for each simulation parameter, for both scalp potentials and SL transformed data.

Note that since the simulations were performed without any noise for the sake of clarity, the obtained results are deterministic, and hence any observed difference (beyond the rounding error due to the temporal sampling rate) is a true difference. Hence, no statistical tests are necessary, nor even possible to perform (since there is no error term).

### 2.2 Simulation # 1

This first simulation illustrates how the recovered timing of scalp potentials is altered by the volume conduction effect, and how the SL transform allows to better recover the underlying generator time courses. To do so, the dipole time courses were manipulated: The right (red) dipole time course, with a peak (mean of the Gaussian) set to 100 ms, and with a spread (standard deviation) set to 180 ms was kept constant, while the peak latency of the left dipole (blue) was varied from 150 to 250 ms, with a constant spread also equal to 180 ms. The amplitude of the dipoles at their peak was 25 mA/m^3^, and kept constant throughout the simulation (Fig. 5A).

#### 2.2.1 Results

We will first describe the case where the two dipoles had the largest temporal difference (100 and 250 ms peak latencies), and the global results for all simulations will be presented later.

**Fig. 3:**
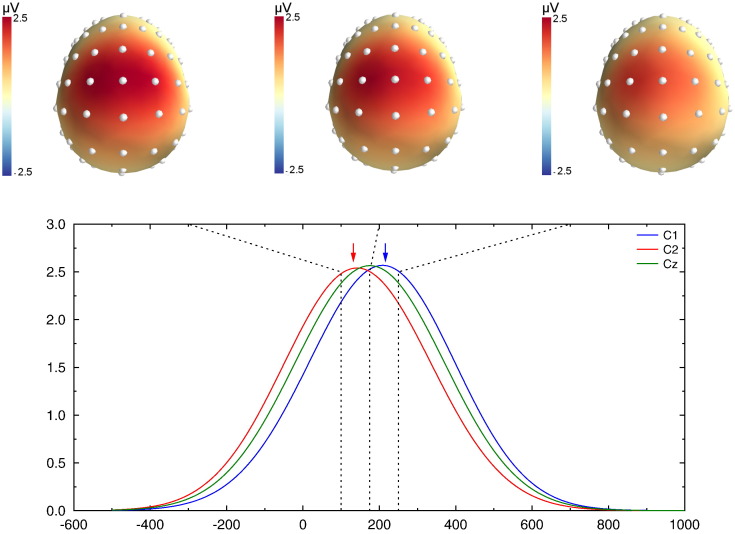
Simulated potentials for the last simulation (100 ms vs. 250 ms). The upper row shows the scalp potential topographies obtained at 150, 175 and 250 ms. Potentials present a large central positivity. Lower panel: time course of the reconstructed potentials at electrodes C1 (blue), Cz (green) and C2 (red). The blue and red arrows indicate the latency of the recovered peak on C1 and C2. The recovered latencies on electrodes are pretty far from the underlying dipole ones. One can also note the large activity observed on Cz, despite the absence of direct underlying dipole.

Fig. 3 shows the recovered scalp potentials for the left mastoid reference (for the sake of clarity, we first restricted analysis to the left mastoid reference since it illustrates the effects common to all reference schemes. A more systematic comparison is presented below). The top panel shows the topographies at the true dipole peak latencies (100 and 250 ms), and at 175 ms, in between the two dipole latencies. Although the activity of two dipoles was generated, the topography shows a largely extended central positivity that does not allow to distinguish these two activities, illustrating the volume conduction effect. The first (left) and last (right) dashed lines in Fig. 3 correspond to the peak latency of the simulated dipoles. The comparison between the dashed lines and the peaks of the recovered activities (colored arrows) indicates that latencies of the scalp peaks do not correspond to the peak of the underlying dipoles. Indeed, the latency measured by the electrode located over the earliest dipole is overestimated (by 44 ms), and the one over the second dipole is underestimated (by 33 ms). As a consequence, while the true timing difference is 150 ms, the observed one is only 73 ms, barely a bit more than half of the real value.

**Fig. 4:**
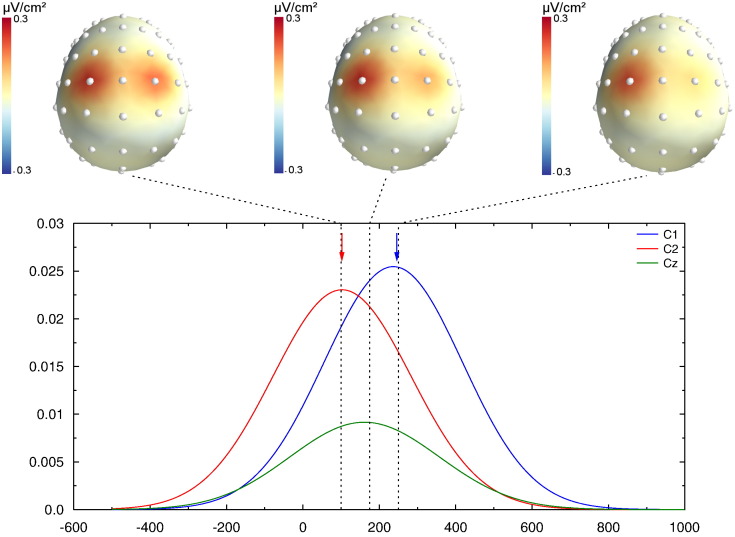
CSD transform of the data presented in Fig. 3. The topographies (upper row) are much better resolved, with two clear peaks of activity above the underlying dipoles. As indicated by the two colored arrows, the peak latencies of the recovered CSD nicely fit the underlying dipole peak. The recovered activity over Cz is of much lower amplitude than on C1 and C2.

Fig. 4 shows exactly the same data, after SL transform computation. They differ from the potential in two main aspects. First, the spatial resolution of the topographies is clearly different: instead of obtaining a large positivity, two independent loci can be observed, each one in close vicinity to electrodes C1 and C2, located above the simulated dipoles. Relatedly, while the amplitude of the potential obtained at electrode Cz was comparable to C1 and C2 electrodes (see green trace in Fig. 3), this amplitude is dramatically decreased after SL computation (see green trace in Fig. 4). Second, the timing of the CSD activities also largely differs: the first and last dashed lines, indicating dipole peaks, are now aligned with the peak of the CSD activities on C1 and C2 (colored arrows), indicating that the CSD activities peak approximately at the latencies of the underlying simulated sources.

**Fig. 5:**
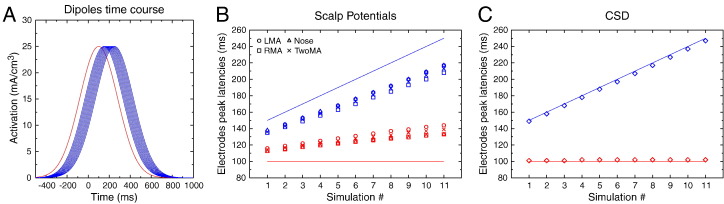
A. Time course of the simulated cortical dipoles. B. Peak latencies of the simulated dipoles (solid lines), and recovered potential peak latencies for each reference frame (LMA: left mastoid; RMA: right mastoid; TwoMA: linked mastoids). It is clear that the latency of the right (earliest, in red) dipole is overestimated, while the latency of the left (latest, in blue) one is underestimated, whatever the reference. C. Same information after CSD transform: The recovered latencies are in close agreement with the underlying dipole time courses.

To visualize how scalp potentials and CSD data differ, we extracted the latency of the peak of activity on C1 and C2 electrodes for the different simulated time courses. Fig. 5B shows the actual dipole latencies (solid lines) and the recovered latencies (open symbols) at scalp level, for each reference electrode configuration. Whatever the reference, the potentials recorded at C1 (blue symbols) clearly underestimate the peak latency of the underlying dipole, while the potentials obtained at C2 (red symbols) overestimate the underlying dipole peak latency. This convergence of the two time courses at scalp level is a typical example of the mixture effect induced by volume conduction. While the dipole latency differences varied between 50 and 150 ms, the scalp potential differences only varied between 20 and 80 ms. The time distortion induced by this mixture is hence pretty large and the recovered difference is around half the value of the true difference (note that the exact value largely depends on the dipole configuration and time courses, and cannot be taken as a general rule). Interestingly, the overall distortion pattern is present whatever the reference electrode. Some small differences appear, however, that deserve comments. The temporal distortion is lower for the electrodes ipsilateral to the reference (see blue circles and red squares) and greater for the electrodes contralateral to the reference (blue squares and red circles). This exemplifies how the reference acts as a weighting factor in the mixture of activities. The linked mastoids present a compromise between the two distortions, leading to values in between the two lateral ones. Based on data presented in Fig. 5B, the Nose reference may look like the one introducing the smallest amount of distortion. Note, however, that this is only due to the specific dipole configuration used in the present example (two lateral ones) that amplifies the effect of lateralized references. If the same simulation is performed with two dipoles in the antero-posterior axis, the Nose reference creates more distortion than the mastoid ones (data not shown).

A completely different pattern is obtained after SL computation (Fig. 5C). The CSD peak latencies almost perfectly fit the peaks of the underlying dipoles: in the largest difference case (dipoles at 100 and 250 ms), the recovered latencies are 103 and 246 ms, respectively, namely a difference of 144 ms compared to the true 150 ms. Note that, since CSD is reference-free, the obtained values are exactly the same whatever the reference used for scalp potentials.

#### 2.2.2 Discussion

Through this first simulation study, we illustrated how volume conduction and, to a lesser extend, reference electrode can affect the temporal resolution of EEG: by systematically varying the time course of one of two simulated cortical dipoles and measuring the recovered scalp potentials and CSD time courses, we showed that volume conduction makes the two time series converge toward each other, hence leading to an overestimation of the latency of the earliest activity, and an underestimation of the latest one. These results illustrate how volume conduction effects, not only blur the spatial resolution of EEG, but also dramatically degrade its temporal one. Accordingly, increasing the spatial resolution by removing (a large part of) the volume conduction effects, largely improves the temporal resolution of the signal.

This first simulation study showed how volume conduction induces a temporal mixture of the cortical sources and hence can mask, at scalp level, underlying real source chronometric differences. In the second simulation, we will see that the same mixture effect can artifactually create scalp latency differences when there is none on the underlying brain sources.

### 2.3 Simulation # 2

Even if volume conduction decreases the temporal separability between brain sources, it is usually considered that when a latency difference is observed on the scalp, it can safely be interpreted as reflecting a true chronometric difference in brain activation. This second simulation will, unfortunately, show that is not the case neither.

**Fig. 6:**
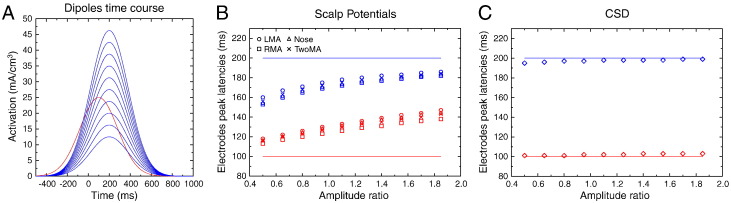
A. Time courses of the simulated cortical dipoles. B. Peak latencies of the simulated dipoles (solid lines) for each dipole–amplitude ratio, and recovered potential peak latencies (empty symbols) for each reference electrode. Despite a constant time course of the underlying dipoles, the recovered scalp latencies differ dramatically as a function of the amplitude ratio. C. Same information after CSD transform: The recovered latencies are in close agreement with the underlying dipole time course.

In this second simulation, we used the same dipoles as above (see Fig. 2). The first dipole had the same time course as in the first simulation (peak latency = 100 ms, peak amplitude = 25 mA/m^3^, kept constant), and the second had a time course peaking at 200 ms. Those latencies were kept constant across all conditions. We, however, varied the relative amplitude of the two dipole activities from 0.5 to 2 (see Fig. 6A). All other parameters were the same as in simulation # 1. As we will see, although the dipole peak latencies are kept constant, the reconstructed scalp potentials will be biased toward either the early or the late source latency, depending on the relative source amplitudes, hence inducing artifactual chronometric differences.

#### 2.3.1 Results

Fig. 6B shows the recovered scalp potential peak latencies at electrodes C1 and C2, as a function of the ratio between the two dipole amplitudes. Let's remind that the latencies of the two dipoles were kept constant (solid lines), and only the amplitude of the second dipole was varied. The latency of the earliest activity is systematically overestimated, while the latency of the latest one is underestimated, as already shown in the first simulation. More importantly, the actual recovered latencies largely depend on the amplitude ratio: the two peak latencies are always biased toward the largest source. Hence, varying only the amplitude makes the latency to artifactually vary.

As for the first simulation, the choice of the reference slightly modulates the latencies, but the overall pattern is the same.

Again, a completely different pattern of results is obtained after SL computation: when the relative strength of the two dipoles is varied, the recovered SL timing does not vary and is weakly affected by the large amplitude change in dipole activity (Fig. 6C). The SL hence allows to much better recover the true underlying dynamic, avoiding erroneous interpretations.

#### 2.3.2 Discussion

The present simulation exemplifies how volume–conduction-induced scalp mixture can lead to incorrect conclusions about the underlying source dynamic. Indeed, while the source time-courses were kept constant, their (relative) amplitude dramatically affected the recovered scalp potential chronometry: increasing the “late” source amplitude induces a global latency increase of the scalp potentials, for all recording electrodes. What would be the functional consequences of such a latency increase? Let's consider that the leftmost (x = 0.5) and rightmost (x = 1.9) data in Fig. 6B correspond to two experimental groups *A* (control group) and *B* (patient group). Based on the observed shift in latencies, widely present, one would certainly conclude that processing speed was reduced in group *B* compared to group *A*. Such conclusions on the impact of pathology on brain processing would definitively be incorrect. Indeed, the two groups actually present a perfectly similar time course, and the increased response is not at all general, but very limited to a single brain region.

SL transformation prevents this incorrect interpretation of the data by recovering the actual latencies, and would have led to the correct conclusion that patients in group *B* do not present any speed deficit, but instead a relative decrease/increase in activity on some specific areas.

### 2.4 Interim discussion of simulations

In the two reported simulations, we showed how volume conduction can hide (or at least severely reduce) real brain timing differences, but also artifactually create false timing differences. More specifically, the simulations show three key aspects: i) the recovered scalp potential time-courses are only poorly related to the true underlying brain source dynamics, ii) scalp potentials tend to largely underestimate temporal differences between brain sources and iii) apparent changes in latencies between experimental conditions/groups do not necessarily reflect an underlying change in timing, as changes in amplitude of the brain generators can induce artifactual latency changes. Hence, while EEG is often promoted for its excellent temporal resolution, the present simulations show that the actual temporal resolution based on scalp potentials is much lower than classically assumed. Importantly, this degraded temporal resolution stems from the same factors affecting the spatial resolution of EEG, mainly volume conduction and reference electrode. As a consequence, improving the spatial resolution of EEG in turn also improves its temporal one.

As a matter of fact, temporal distortions disappeared after SL computation, and the timing of the recovered activities is much more similar to those of the sources than the scalp potentials. In other words, it is only after having improved its spatial resolution that EEG really reaches a good temporal resolution.

Although we used a realistic head model (geometries and conductivities), the main purpose being illustrative, we used simplified time courses (only two dipoles, smooth time courses, no noise etc.). Therefore, one may argue that 1) the convergence evidenced here is not (or less) present with realistic signals and/or 2) under more realistic conditions, the improvement induced by CSD is much less than in such ideal situations. In what follows, we will show the same type of effects on real data, in two different conditions (response-related vs. stimulus-related activities) and with two different ways of computing CSD (the so-called “source derivation” method, Hjorth, 1975; MacKay, 1983 vs. spline interpolation approach, Perrin et al., 1987; Kayser and Tenke, 2006b).^3^

## 3 Empirical data

### 3.1 Dataset # 1: deciding and acting

The first dataset comes from Vidal et al. (2003) and concerns cortical processes involved in response selection and execution. This study was interested in the functional organization of the pre-motor (mainly the pre-supplementary motor area, pre-SMA) and motor (mainly primary motor areas) areas in selecting and executing a response in bimanual choice situation.

#### 3.1.1 Stimuli and task

All details about this dataset can be found in Vidal et al. (2003). Only the relevant information will be described here. Participants performed a manual Stroop task, in which they had to respond with a right or a left hand key-press as a function of the color of a written word. Responses were given by thumb presses, and electromyographic (EMG) activities of the two *flexor pollicis brevis* were measured. Scalp potentials were referenced to the left mastoid. After careful artifact rejection, the data were averaged time-locked to EMG onset. EEG was recorded with 21 scalp electrodes, positioned so that the SL could be estimated by the source derivation method (Hjorth, 1975), as modified by MacKay (1983). With such method, the SL at electrode *O* is computed as [3*V*_*O*_ − (*V*_*A*_ + *V*_*B*_ + *V*_*C*_)]/*d*^2^ where *V_O_*, *V_A_*, *V_B_* and *V_C_* represent the potential recorded at electrodes *O*, *A*, *B* and *C*, provided that electrode *O* is at the barycenter of the triangle *ABC*, at a distance *d* of each vertex.

For the sake of simplicity, right and left responses were collapsed, after having mirrored the activities for the left response (C4 for the left response was combined with C3 for the right response). Hence, C3 in fact reflects the activity of the electrodes located above the M1 contralateral to the executed response.

#### 3.1.2 Results

**Fig. 7:**
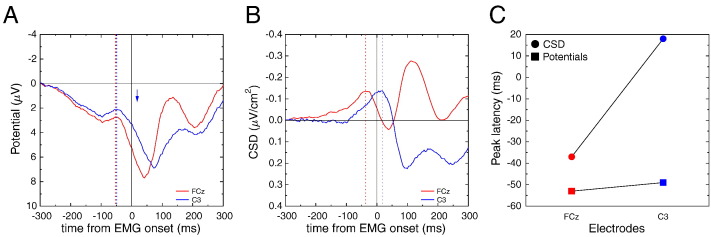
Empirical data of dataset # 1: A: scalp potential time course recorded by electrodes C3 and FCz in a manual reaction time task (right response), time-locked to the response-EMG onset. The time courses are pretty similar between the two electrodes, and mainly present a large and temporally spread positivity that peaks slightly after EMG onset. A small bump is observed around − 50 ms, with a similar time course for both electrodes. B. Same data after SL computation: The recovered activities dramatically differ between the two electrodes. Over electrode FCz, a negative peak is observed around 40 ms before, and resolves shortly after, EMG onset. Over C3, a negative activity peaks shortly after EMG onset (around 20 ms). Panel C summarizes the obtained latencies (y-axis) as a function of the electrodes (x-axis, red: FCz, blue: C3), for scalp potentials (colored squares) and CSD (colored circles).

Fig. 7A plots the scalp potential data recorded at electrode FCz and C3 (reference: left mastoid), time-locked to EMG onset. The time courses mainly reveal large positive components peaking just after EMG onset, and hence pretty close to the response onset, as already revealed by Jung et al. (2001). One can also detect, however, small negative bumps around − 50 ms. The two electrodes present very similar time courses, with peaks of activity very close temporally. Fig. 7C plots the peak latencies (square symbols for scalp potentials). SL data (panel B) provide a very different view: the activity obtained at FCz peaks at − 37 ms, clearly before the peak observed on C3, at + 18 ms (see circle symbols in panel C). The two SL time courses thus present a clear sequential activation, with FCz activity starting, peaking and ending earlier than C3 activity. This statistical difference in latencies was assessed in the original article (Vidal et al., 2003), and the reader is referred to this article for details. For the sake of comparison, the latency of the CSD peak over C3 is reported (blue arrow) on the scalp potential traces. No noticeable event occurs at this time range on scalp potential data.

#### 3.1.3 Discussion

As in the simulation study, scalp potentials recorded in this experiment show similar time courses for both FCz and C3 electrodes (about 7 cm apart). After CSD computation, however, a different pattern is observed: the activity of the two electrodes is separated temporally, revealing two cortical generators, activated sequentially. Thus, as in the previous simulations, the presented data show that volume conduction effects hinder timing differences between brain regions. Applying SL transform to the data reveals much larger temporal differences which, according to the simulations above, likely reflect true underlying brain activity differences.

From a functional point of view, the interpretation based on SL supports a sequential involvement of the (pre)SMA and M1 in the selection and execution of response (Vidal et al., 2003, 2011; Burle et al., 2004), at best scalp potentials do not allow to reach this conclusion, and at worse, one may conclude, based on the similar time courses, that the underlying cortical areas work in parallel.

In the present dataset, CSD was approximated by the source derivation method (Hjorth, 1975; MacKay, 1983). Since this original publication (Vidal et al., 2003), this sequential activation has been replicated several times with both Hjorth method (e.g. Vidal et al., 2011) and with the spline interpolation one (see e.g. Carbonnell et al., 2013). In some of those studies, these two activities were shown to be independently modulated by different factors, which confirm that they reflect the activation of independent cortical generators.

Note that the separation of such activities, providing powerful markers of response selection and execution, was never possible on the scalp potential data.

### 3.2 Dataset # 2: dynamics of visual perception

The second dataset comes from a study by Burle et al. (2008). In the original publication analyses were concentrated on response related activities. In the present context, we will focus on visual evoked potentials (VEPs), which were not reported in the original paper.

#### 3.2.1 Stimuli and task

The details of the task can be found in Burle et al. (2008), and only the relevant aspects will be presented here. Subjects performed an Eriksen flanker task (Eriksen and Eriksen, 1974), in which stimuli were composed of 3 letters that were either identical (e.g. *HHH*, compatible stimuli), or with the lateral letters differing from the central one (e.g. *SHS*, incompatible stimuli). The stimuli were presented centrally. Participants had to respond with a left or right hand key-press as a function of the nature of the central letter (for example, respond “left” to a central *H* and “right” to a central *S*).

#### 3.2.2 EEG acquisition

EEG was acquired with 64 active-2 electrodes (Biosemi, Amsterdam) located at the standard extended 10–20 system. All electrodes were off-line referenced to the left mastoid. After ocular artifact correction (Gratton et al., 1983), all the signals were carefully inspected to remove all other artifacts. Great care was taken to remove local artifacts, since CSD computation is very sensitive to them. For the current purpose, all the non-rejected trials were averaged time-locked to the stimulus onset, and visual evoked potentials were analyzed. The parameters for CSD computation are the same as for the simulations presented above.

#### 3.2.3 Results

**Fig. 8:**
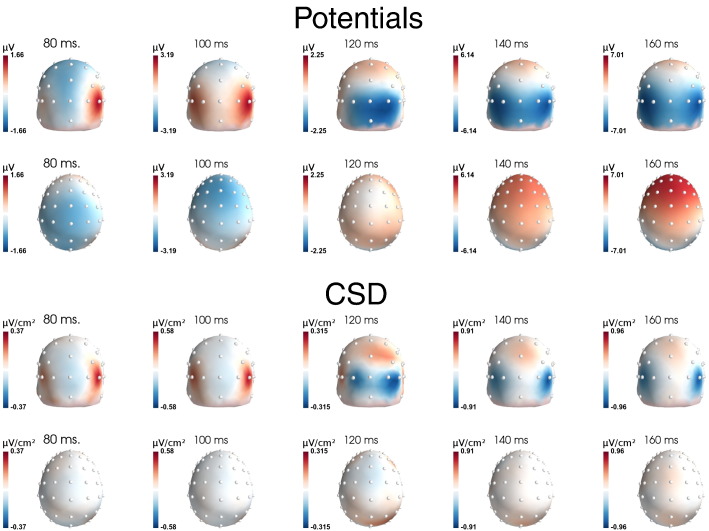
Topographies obtained on dataset # 2. The first two rows present the scalp potential topographies, viewed from back (first row) and top (second row), from 80 to 160 ms. The two lowest rows present the same data, at the same latencies, after CSD computation.

Fig. 8 shows the topographies of the visual evoked potentials (VEPs) at different time points, for both scalp potential data (first two rows), and after CSD transform (last two rows). As expected, CSD maps show more focal activities than potential topographies, as shown for the occipital and parietal zones (first and third row). For instance, comparing activities at 120 and 140 ms on CSD maps reveals a flow of activity from medial toward lateral electrodes. Although this flow is also partly seen on potential data, it is much less clear. The second row reveals another interesting pattern: while the scalp data show pretty large voltage activities on frontal electrodes (first negative — 80 and 100 ms, then positive — 140 and 160 ms), those activities are absent on CSD (fourth row). Such frontal “activities” actually reflect activations generated in occipital and parietal regions that are volume conducted to the frontal electrodes.

**Fig. 9:**
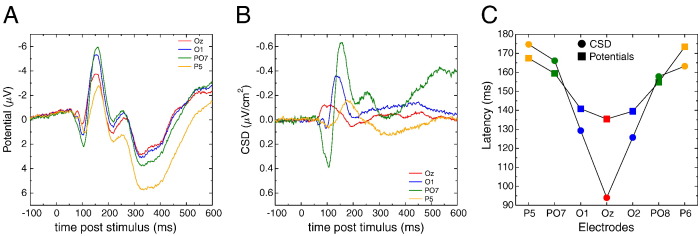
Time courses obtained from dataset # 2. A. Time course of the negative scalp potential at the selected occipital and parietal electrodes. Only the left electrodes are shown. Activities over the different electrodes present a very similar shape and time course. B. Same data after SL transform: the shape and the timing of the activities dramatically differ between electrodes. C. Summary of the peak latencies (y-axis) for the representative electrodes (x-axis) for scalp potentials (colored squares) and CSD (colored circles). While the latency differences are rather small for scalp potential data (max difference: 38 ms), the CSD evidences a clear occipito-parietal latency gradient (max difference: 81 ms).

Fig. 9A presents the VEP time courses for some representative occipital and parietal electrodes (Oz, O1, PO7 and P5, a very similar pattern is observed at homologous sites over the right hemisphere, see panel C). On the scalp potential VEP, one can observe the “standard” components: P1 (around 100 ms), N1 (around 150 ms) followed by N2 (around 250 ms) and finally a large P3 (around 350 ms). These components, present in a large proportion of electrodes, peaked at pretty similar latencies although a gradient exists from medial to lateral electrodes (from earliest to latest response: 38 ms difference, see squares on panel C).

Panel B of Fig. 9 presents the very same data and electrodes, after CSD transform. A very different pattern can be observed. First, while scalp potential data show a highly correlated time course across electrodes, here the time courses are dramatically different from one electrode to the other. More importantly, this translates into different latencies of the peaks of these activities (from earliest to latest: 81 ms difference, see circles on panel C of Fig. 9). To assess the difference in timing between scalp potential and SL data, we ran an ANOVA including 7 sites^4^: electrodes P5, PO7, O1, Oz, O2, PO8 and P6 and measure type (scalp potentials vs. SL) as within participant factors. These analysis revealed a main effect of electrodes (*F*(6, 54) = 14.96, *p* < .001, *ε* = 0.43) and no main effect of measure type (*F*(1, 9) = 1.72, *p*= .22). Importantly, the interaction between the two factors was significant (*F*(6, 54) = 2.48, *p* < .04), confirming that the latency gradient was larger after SL computation.

#### 3.2.4 Discussion

Sensory-evoked potentials have been much more studied than response-related components with EEG. In most studies, only scalp potentials were analyzed. The present data confirm that for visual stimuli, by improving the spatial resolution of EEG (Fig. 8), one can reveal a consistent ordering of activities flowing from postero-medial toward antero-lateral electrodes, and likely corresponding to different functional visual processes (see e.g. Riés et al., 2013; Fahrenfort et al., 2007; Foxe and Simpson, 2005). How to functionally and physiologically interpret those various activities is arguably beyond the scope of the current paper, and would require extensive work.

One can note, however, that because of this large span of latency peaks, one may wonder what “the” N1 observed on scalp potential really means, as it clearly reflects a compound of sources, which mixes several functionally very different brain activities. Careful examination of those different activities would undoubtedly provide a much more detailed description of visual processing.

## 4 General discussion

Electroencephalography is one of the few techniques allowing to non-invasively study brain functioning with a timing that (potentially) matches the one of the processes under investigation, that is, in millisecond range. Being largely portable, it further allows a very flexible use, making it unique and, despite being one of the oldest imaging techniques, it remains a promising one for the future. Its main limitation is often considered to be its low spatial resolution, its main strength being its “excellent” temporal one. In the present report, we argued that both these strengths and weaknesses are overestimated. As a matter of fact, it has long been argued that the spatial resolution of EEG can easily be improved by estimating the scalp CSD (see Babiloni et al., 2001 for an historical perspective, Tenke and Kayser, 2012; Giard et al., 2014 for recent overviews): while the scalp potential spatial resolution is usually considered to be around 6–9 cm (Babiloni et al., 2001), CSD estimation allows to reach a spatial resolution of 2–3 cm, which comes close to the size of brain areas. On the other hand, despite the largely accepted idea that EEG has an excellent temporal resolution, the actual temporal resolution of conventional scalp potential EEG is lower than usually thought as the factors degrading the spatial resolution of EEG (mainly volume conduction and reference electrode) also degrade its temporal one. Importantly, having common origins, improving the spatial resolution mechanically ameliorates the temporal grain of EEG. Said differently, in order for EEG to reach a real good temporal resolution, it is necessary to amend its spatial one.

In the first part of this work, two simulation studies illustrated this interrelationship between spatial and temporal resolutions by showing how volume conduction not only spatially blurs the underlying brain signals, but also *temporally* distorts their recovered scalp counterparts: because of the spatial smearing, the time course of the recovered scalp potentials is a mixture (i.e. weighted sums) of the true underlying source time courses. For this reason, the scalp potentials recorded at different electrode locations present peaks of activity at latencies that are intermediate between the true latencies of the neural event peaks. As a consequence, the timing of scalp potential peaks of activity does not generally correspond to underlying cortical sources peaks, and correlatively, at the moment of source peak activity, there is not necessarily a peak of activity in the scalp potentials. It is hence not safe to infer the timing of brain events based on the scalp potentials. Critically, by spatially deblurring the scalp recorded activities, SL also *temporally* unmixes the recovered time courses and provides a much better estimate of the underlying neural event peaks. Indeed, the latencies of the peak of the CSD estimates at electrodes in the vicinity of the underlying cortical sources^5^ nicely fit with the latencies of activity of the cortical sources. Inferring timing of brain events based on the SL transform is hence much reliable than on scalp potentials. Note, however, that while SL dramatically reduces the problem, it does not necessarily solve it entirely: for spatially very close sources, that is below the spatial separability of the SL, the recovered CSD activities will still be a mixture, and the timing still be biased toward the largest source. But, as far as SL can spatially separate sources, it will provide a better temporal resolution than scalp potentials. Another issue might be the spatial sampling (i.e. the number of electrodes). However, Kayser and Tenke (2006b) have reported that the CSD reconstruction with low density estimates is very good approximations of high density estimates.

Although the first simulation showed that one should not infer the absolute timing of brain events from the scalp potentials, one could at least hope to be able to infer the *relative* timing between experimental condition and/or populations. The second simulations show that even such conclusions on the relative timing should be drawn with caution. Indeed, since the scalp potentials are a weighted sum of the underlying sources, if the relative strengths of early and late sources change, this produces *global* shifts in latency, over all electrodes, mimicking a chronometric difference in the sources time courses. An apparent change in latencies does hence not necessarily reflect a true chronometric difference. Again, CSD estimation removes this ambiguity as the recovered time courses are not (or at least much less) affected by remote sources, and are hence not biased toward the largest source.

In the simulations, realistic head model (geometries, conductivities etc.) was used. However, for the sake of clarity and to better illustrate the volume conduction and reference electrode effects, we restricted the simulations to only two dipoles, with very simple (i.e. very smooth) time courses. To generalize the results, similar effects were then shown on two empirical data sets: one related to response selection and execution, and the second one related to visual information processing. Those two datasets were also chosen because they are based on two different methods to compute SL, hence evidencing the robustness of the method.

In both datasets, while scalp potentials presented very similar activities (in terms of shape, timing etc.) on different electrodes (up to 7 cm), the SL data provide a very different view: CSD activities differ dramatically, even at close-by electrodes (Oz and O1/O2 are separated by only 2.5 cm), with very different time courses and morphologies. In both cases, SL allowed to reveal specific activities that were not suspectable on the scalp potentials.

In the first empirical case described above (Section 3.1), the timing of the peak of the small negative bump (around − 50 ms, dotted colored lines) does not correspond to any local underlying events (compare with dotted colored lines in Fig. 7B). Reciprocally, while CSD reveals a peak of activity around 20 ms post-EMG over the contralateral M1, no clear electrical event can be detected at that time on scalp potentials (position of the blue arrow in Fig. 7A), and one would be tempted to consider that no significant brain event occurred in this latency range, which would be clearly wrong. Hence, contrary to what is often assumed, the scalp potentials do not provide an adequate temporal description of brain activity. From a functional point of view, since the activities recorded by electrodes FCz and C3 are very similar, one may conclude that the underlying generators have similar time courses. However, this hypothesis is clearly rejected after CSD computation, since the time courses for electrodes FCz and C3 get dramatically different, and evidence a sequential activation, likely of SMA/pre-SMA and M1. These data show that a true difference in timing can be completely hindered by the mixing effect of volume conduction. Although it is well known that the null hypothesis can never be accepted, it is interesting to note that in the present example, CSD avoids this pitfall by allowing to reject it.

The second dataset evidences similar type of effect on visual evoked potential. Scalp potentials have a very similar shape and timing over different occipito-parietal electrodes. Indeed, all (represented) electrodes first present an early positivity around 100 ms, followed by a negativity around 170–200 ms and a later large positivity after 300 ms. While the timing of the negative peak varies slightly across electrodes (less than 40 ms, see Fig. 9C), the scalp potentials appear largely driven by the (rather) late occipito-parietal activities evidenced on electrodes PO7/PO8 after CSD (green trace in Fig. 9B). As a consequence, the latencies observed on more medial and more posterior electrodes (e.g. Oz, O1/O2) are overestimated, being attracted by the large occipito-parietal one. Again, CSD reveals clearly different activities, with different locations, timing and shapes, very likely originating from different cortical structures (see Riés et al., 2013), and corresponding to different functional visual processes, opening new perspectives, both in terms of cognitive interpretation (see e.g. Fahrenfort et al., 2008) or in terms of pathologies (see e.g. Kayser et al., 2012).

A last comment is in order: it is usually considered that CSD “simply” improves the spatial resolution of EEG, and hence that we see the same things, but better. By evidencing that the scalp potential time courses do not correspond to the underlying brain sources, and that CSD allows to much better recover the true time course, CSD actually allows to reveal *new components*, not suspectable on scalp potentials. Hence, CSD does not “merely improve” EEG, but actually provides a pretty different, and, we believe a much more accurate, view of the true underlying brain activities. This has been very clear for response monitoring, where SL transforms allowed to reveal that a specific activity occurring just after an incorrect response (the so-called “error negativity” — *N_e_*, Falkenstein et al., 1991, or “error related negativity” — *ERN*, Gehring et al., 1993), was not specific to errors. Indeed, while no such equivalent activity was visible on correct trials with scalp potential, SL transform revealed the existence of a similar wave, of lower amplitude though, on such trials (Vidal et al., 2000, 2003; Roger et al., 2010, see also Kayser and Tenke, 2006a,b for similar effects in a different context), which has recently been confirmed by intra-cerebral recordings in Humans (Bonini et al., 2014).

## 5 Conclusions

It is classically accepted that computing the SL of the scalp potential data improves the spatial resolution of EEG. Here we show that it also improves its temporal resolution, which is actually overestimated for the conventional EEG, that is scalp potentials. Indeed, while the theoretical temporal resolution of EEG is excellent, its actual one is lowered by the very same physical phenomena degrading its spatial resolution. Improving the second one, mechanically improves the first one. We also showed that the degraded spatio-temporal description of the underlying phenomena actually leads to incorrect inferences about brain functions. A widely encountered argument in the EEG community is that being interested only in the timing of brain activities, without necessarily determining where those activities come from, one does not have to be too concerned by the bad spatial resolution of EEG. We have shown that such argument is not valid, as the timing of the low spatial resolution scalp potential is distorted and hence provides an incorrect description of brain activity time courses. It hence appears essential that the EEG community more systematically uses techniques allowing for better separating brain sources. Among the possible techniques, SL remains a very interesting and powerful candidate, as it provides a remarkable spatial and temporal improvement at limited costs, both in terms of computation and assumptions.

## Footnotes

1 A third type of technique can also be considered as imaging: the stimulation or interference based techniques, such as transcranial magnetic stimulation — TMS. Such interference techniques will not be covered here.

2 With the 100 ms delays simulated, and with a “temporal resolution” (TR) parameter set to 1 s, the simulated haemodynamic response are *exactly* the same. To see a difference appearing, one needs to assume an unrealistic TR of 300 ms or less.

3 The source derivation method and the spline interpolation approach have been shown to provide highly comparable results (Tandonnet et al., 2005; Tenke et al., 1998).

4 In case of sphericity violation (assessed by Mauchly's test), Greenhouse–Geisser correction was applied. Note that the same results were obtained after Huynd–Feldt correction.

5 It is important to note that the peak of activity is not necessarily right above the source. This is especially true for tangential dipoles, where the peaks of activity – positive and negative – will be symmetrical around the true position of the dipole (see Giard et al., 2014 for example in the auditory domain). However, dipoles are rarely purely radial, and hence also own a tangential component. In such case, the tangential component also shifts the scalp topography.
